# Primary Nonfunction of Renal Allograft Secondary to Acute Oxalate Nephropathy

**DOI:** 10.1155/2011/876906

**Published:** 2011-09-28

**Authors:** Ravi Parasuraman, Ping L. Zhang, Dilip Samarapungavan, Krishna Pothugunta, Gampala Reddy, Leslie Rocher, Francis Dumler, Vandad Raofi, Steven Cohn, Alan Koffron

**Affiliations:** ^1^Division of Nephrology and Transplantation, William-Beaumont Hospital, Royal Oak, MI 48037, USA; ^2^Kidney Transplant Outreach Program, William-Beaumont Hospital, Royal Oak, MI 48037, USA; ^3^Oakland University, William Beaumont School of Medicine, Royal Oak, MI 48037, USA; ^4^Division of Renal Pathology, William-Beaumont Hospital, Royal Oak, MI 48037, USA

## Abstract

Primary nonfunction (PNF) accounts for 0.6 to 8% of renal allograft failure, and the focus on causes of PNF has changed from rejection to other causes. Calcium oxalate (CaOx) deposition is common in early allograft biopsies, and it contributes in moderate intensity to higher incidence of acute tubular necrosis and poor graft survival. A-49-year old male with ESRD secondary to polycystic kidney disease underwent extended criteria donor kidney transplantation. Posttransplant, patient developed delayed graft function (DGF), and the biopsy showed moderately intense CaOx deposition that persisted on subsequent biopsies for 16 weeks, eventually resulting in PNF. The serum oxalate level was 3 times more than normal at 85 **μ**mol/L (normal <27 **μ**mol/L). Allograft nephrectomy showed massive aggregates of CaOx crystal deposition in renal collecting system. In conclusion, acute oxalate nephropathy should be considered in the differential diagnosis of DGF since optimal management could change the outcome of the allograft.

## 1. Introduction

Primary Nonfunction (PNF) defined as permanent loss of allograft function starting immediately after transplantation accounts for 0.6% to 8% of all renal graft loss and is significantly associated with poor patient survival [1–4]. The causes of early allograft dysfunction are changing constantly, and recently calcium oxalate (CaOx) crystal deposition has been added to this list. Deposition of CaOx crystal in renal tubules can be seen in >50% of allograft biopsies performed <3 months posttransplant [5]. Although presence of CaOx crystal in allografts can be benign, when present in moderate intensity, it contributes to increased incidence, acute tubular necrosis and poor allograft survival [5, 6]. Here we present a case of extreme complication of CaOx crystal deposition resulting in primary nonfunction of renal allograft that has not been previously reported to our knowledge.

## 2. Case Report

A-49-year old male with end stage renal disease (ESRD) secondary to autosomal dominant polycystic kidney disease (ADPKD) with past medical history of hypertension, hypothyroidism, secondary hyperparathyroidism, and polycystic liver disease underwent a 6 antigen mismatch, expanded criteria donor (ECD) kidney transplantation. The panel reactive antibody (PRA), donor-specific antibody (DSA), and flow crossmatch (FCX) were negative prior to transplantation. Donor information includes a 75-year-old female with serum creatinine (Scr) of 0.5 mg/dL (44.2 *μ*mol/L) and urine output of 400 mL/hour with negative urinalysis prior to organ recovery. The pre-implant renal biopsy showed 3% glomerulosclerosis without interstitial inflammation or fibrosis. The kidney was on simple cold storage with cold ischemia time of 18 hours. No past history of renal stones or hypercalcemia either in the donor or in the recipient.

## 3. Posttransplantation Course

**Figure 1:**
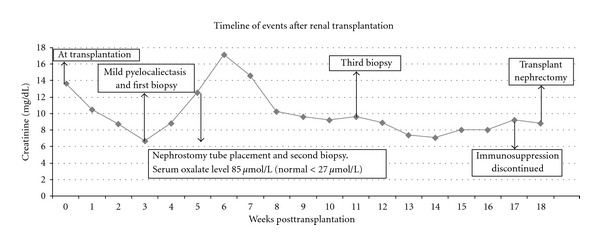
Serum creatinine levels and related events during the course of renal transplantation.

The patient received alemtuzumab (anti-CD52 antibody, Campath, Genzyme Corporation) for induction and tacrolimus and mycophenolate mofetil (MMF) without steroids for maintenance. Other medications included levothyroxine, furosemide, sulfamethoxazole/trimethoprim (Bactrim), and valganciclovir hydrochloride (Valcyte) with dose adjusted for renal function. Posttransplant, the patient developed delayed graft function (DGF) and slow recovery of allograft function with urine volume reaching 2800 mL/day with corresponding decrease in Scr from 13.62 mg/dL (1204.01 *μ*mol/L) to 6.70 mg/dL (592.28 *μ*mol/L) over 2-3-week period. The timeline of clinical events is shown in Figure 1.

**Figure 2:**
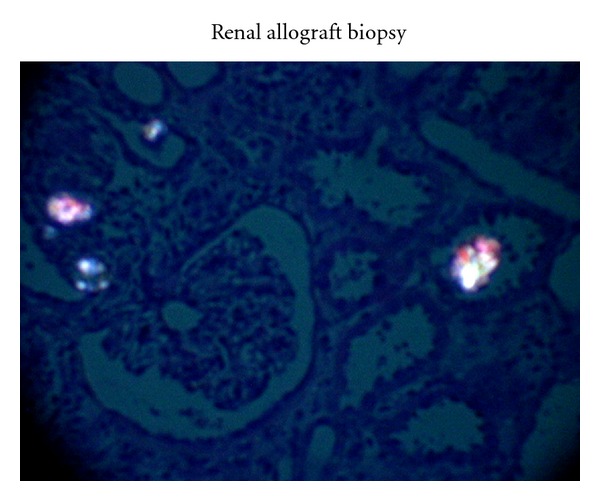
Biopsy Shows calcium oxalate crystal deposition in the renal tubules under polarized light microscopy (1st biopsy performed during 3rd week of transplantation).

**Figure 3:**
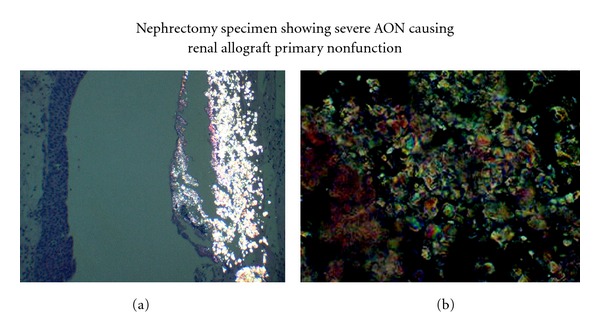
(a) shows widespread massive aggregate of CaOx crystals in the renal collecting system extending into the parenchyma seen under polarized light. (b) shows a region of (a) under high power showing accumulation of polygonal crystals with multicolored birefringence under polarized light. AON: acute oxalate nephropathy, CaOx: calcium oxalate.

An allograft ultrasound (US) and biopsy performed at 3 weeks for slow recovery showed minimal pyelocaliectasis with normal resistive indices (RI; 0.45–0.62) and perfusion. The biopsy was significant for acute tubular necrosis (ATN, confirmed by kidney injury molecule-1 immunostain) and CaOx crystal deposition with no evidence of rejection (Figure 2). In view of CaOx deposition, clinical evaluation performed for secondary hyperoxalosis (enteric hyperoxalosis, MMF-induced diarrhea, excessive oral intake of oxalate-containing foods including vitamin C, ethylene glycol intoxication, or use of methoxyflurane) was found negative. The patient continued his immunosuppression with tacrolimus dose reduction for mildly elevated trough levels days prior to biopsy. Despite tacrolimus dose reduction, the patient showed progressive increase in Scr with subsequent development of microscopic hematuria and moderate fluid overload. Reimaging of the allograft at week 5 showed moderate hydronephrosis with normal RI and perfusion. Because of progression of hydronephrosis, percutaneous nephrostomy and antegrade pyelogram were performed and that showed no ureteral obstruction. A simultaneous biopsy continued to show CaOx deposition and ATN in the absence of rejection or BK virus nephropathy. In view of persistent CaOx deposition, serum oxalate level was measured, and it was 3 times more than normal at 85 *μ*mol/L (normal <27 *μ*mol/L). Due to unrelenting ATN, the dose of tacrolimus was reduced by 50% with addition of prednisone 10 mg/day and cautious increase in furosemide dose to augment urine output hoping to decrease supersaturation of CaOx and crystal formation. Unfortunately Scr remained unchanged despite resolution of hydronephrosis and tacrolimus dose reduction. A third biopsy performed was not significantly different from previous biopsies. Since the patient remained rejection-free, a trial of tacrolimus-free immunosuppression with prednisone and mycophenolate mofetil was attempted for the remaining course of transplantation expecting spontaneous resolution of CaOx-mediated renal injury. Unfortunately, the patient was elected to stay on peritoneal dialysis; otherwise, long duration of daily hemodialysis may have reduced the oxalate burden on the allograft. The patient showed no signs of recovery, and we could not justify the continuation of immunosuppression beyond 15 weeks because of 2 episodes of urinary tract infection. Finally with the diagnosis of PNF, allograft nephrectomy was performed at week 17. The explant showed extensive aggregates of CaOx crystals (multicolored birefringence polygonal crystals under polarized light as shown in Figures 3(a) and 3(b)) in the renal collecting system extending to the parenchyma. In order to exclude the donor origin of CaOx deposition, a review of pre-implant biopsy was performed and showed no CaOx deposition and the mate kidney showed good allograft function with nadir Scr of 1.5 mg% (132.6 *μ*mol/L). The diagnosis of acute oxalate nephropathy (AON) was made based on persistent CaOx deposition in the allograft, high serum oxalate level despite extensive deposition within the kidney, and the explant histology showing extensive CaOx crystal formation in the collecting system in the absence of other causes for allograft dysfunction.

## 4. Discussion

This case of PNF from AON is an uncommon and extreme manifestation of hyperoxalosis. The defining element of AON in this case is extensive deposition of characteristic crystals in tubules and in the collecting system (Figures 1 and 2). The polygonal, refractile, multicolored birefringence under polarized light identifies them as calcium oxalate [7]. Uremic oxalosis, the most frequent cause of hyperoxalosis in ESRD is likely in our patient in the absence of other causes of hyperoxalosis [5]. The predisposing risk factors included peritoneal dialysis (poor oxalate clearance) as the mode of renal replacement therapy pretransplant, higher serum creatinine level (13.62 mg/dL) at transplantation, and the donor age of 75 years [8, 9]. Although up to 39% of all nephrolithiasis in ADPKD is of CaOx in nature, it is unclear whether ADPKD is an added risk factor [10]. This case was determined to be primary nonfunction since the patient was peritoneal dialysis dependent during the entire period despite early minimal improvement in allograft function.

Both primary and secondary hyperoxaluria are well-recognized causes for renal failure. Primary hyperoxaluria (PH) causing AON in renal allograft has been reported and that rare possibility could not be excluded in our patient in the absence of mutational analysis [11]. Secondary hyperoxalosis causing allograft dysfunction is increasingly recognized in recent years. Normally, dietary calcium binds to oxalate in the intestine and is excreted as CaOx in the stool. Fat malabsorption decreases the availability of intestinal calcium thus increasing oxalate absorption resulting in enteric hyperoxalosis, the most common cause of secondary hyperoxalosis. Fat malabsorption from pancreatic calcification causing AON with resultant loss of first allograft and delayed recovery in the second allograft has been reported in a patient who underwent two renal transplants over 7-month period [12]. Recently, a case of AON secondary to MMF-induced diarrhea was reported in a simultaneous kidney-pancreas transplant recipient [13]. Excessive intake of vitamin C (8 grams/day) causing secondary hyperoxalosis and dialysis-dependent AON in a transplant recipient was reported by our group [14]. 

Uremic oxalosis of chronic kidney disease results from accumulation of serum oxalate that is 10–30 times above the normal level [15, 16]. Unfortunately, neither hemodialysis nor peritoneal dialysis normalizes Ox level. In a study of 212 recipients with median plasma oxalate level of 35.0 *μ*mol/L (normal: 2.6–11.0) at transplantation, the oxalate level was shown to be negatively associated with absolute GFR and positively to donor age and plasma creatinine at 10 weeks posttransplantation [5, 9]. Excretion of accumulated serum oxalate can cause injury to allograft in the immediate posttransplant period [5, 11, 17]. This may explain the findings of Pinheiro et al. who showed presence of CaOx deposition in 82% and 52.6% of allograft biopsies performed within 3 weeks and 3 months of transplantation [5]. The presence of CaOx deposition in allografts may be nonspecific or truly pathological, and this diagnostic difficulty is not uncommon clinically. Moderate CaOx deposition was shown to be associated with higher ATN (47% versus 24%) in the immediate posttransplant period and poor allograft survival (49.7% versus 74.1%) at 12 years [5]. The intensity of CaOx deposition score proposed by Scheinman et al. adds value in determining pathogenicity [18]. Clinical diagnosis of AON can be made when ATN is associated with moderate to severe CaOx deposition and higher serum oxalate levels in the absence of other causes. Once the diagnosis is made clinically, management strategies may include (1) adequate fluid administration to maintain high urine output to reduce the risk of supersaturation of CaOx [19]. (2) Correction of metabolic acidosis when present to increase urinary citrate and magnesium levels to prevent CaOx crystallization [19], (3) frequent and prolonged hemodialysis treatment to reduce serum oxalate level to near normal levels, and (4) finally, restricting high oxalate-containing diet such as vitamin C treatment of fat malabsorption and diarrhea when present [12, 14]. 

PNF of renal allograft is significantly associated with poor patient survival with hazard ratio of 2.04 and is further supported by the Canadian organ replacement registry report which shows patient survival to be significantly lower when graft loss occurs <90 days as compared to >90 days posttransplantation [4, 20]. In conclusion, our case reiterates the need for continuous evaluation of DGF patients for all treatable causes. CaOx deposition in renal allograft is an important and probably underrecognized cause of DGF that requires adequate awareness with early intervention to improve the allograft outcome.
